# Physicochemical and FTIR-ATR Spectroscopic Characterization of Lipid Deterioration in Raw Horse Mesenteric Fat Under Stress Storage Conditions

**DOI:** 10.3390/biology15141205

**Published:** 2026-07-21

**Authors:** Moldir Nurseitova, Meruert Syzdyk, Xenia Dronova, Elizaveta Chuvashova, Sanimay Koshieva, Yerkin Massanov, Kuanish Syman, Gaukhar Konuspayeva, Bernard Faye, Nurlan Akhmetsadykov

**Affiliations:** 1 “Antigen” Scientific-Production Enterprise LLP, 4 Azerbayev Str., v. Abay, Karasai District, Almaty Region 040905, Kazakhstan; 2 Faculty of Biology and Biotechnology, Farabi University, Al-Farabi Ave., 71, Almaty 050040, Kazakhstan; 3 Department of General Education Disciplines, Faculty of Languages and Humanities, Egyptian University of Islamic Culture Nur-Mubarak, Al-Farabi Ave., 73a, Almaty 050040, Kazakhstan; 4 Center of International Cooperation on Agriculture Research for Development–CIRAD, Livestock Systems and Animal Products Management—UMR SELMET, Campus International de Baillarguet, CEDEX 5, 34398 Montpellier, France

**Keywords:** equine adipose tissue, peroxide value, lipid oxidation, deterioration kinetics, FTIR-ATR spectroscopy, Central Asia

## Abstract

Horse mesenteric fat is a culturally significant and nutritionally valuable food product in Central Asia, yet the kinetics of its oxidative and hydrolytic deterioration under sub-optimal storage conditions remain poorly characterized. Raw mesenteric adipose tissue from ten free-grazing horses in Northern Kazakhstan was subjected to accelerated stress storage at 30 °C and 70% relative humidity for six days, with physicochemical and spectroscopic evaluation on days 0, 3, and 6. Parameters assessed included acid value, peroxide value, refractive index, density, melting point, sensory attributes, and Fourier-transform infrared attenuated total reflectance spectroscopy. The acid value increased markedly from 1.17 ± 0.46 to 32.60 ± 26.18 mg KOH/g by day 6, substantially exceeding the internationally accepted threshold of 2.0 mg KOH/g, while peroxide value rose progressively and density declined. Melting point decreased significantly between days 0 and 3, a physicochemical change that may be associated with hydrolytic and oxidative modification of the lipid matrix, as corroborated by infrared spectral analysis. These results demonstrate that raw horse mesenteric fat is highly susceptible to rancidity, reaching unacceptable quality thresholds within six days, underscoring the urgent need for cold-chain management and evidence-based handling guidelines for this product across the region.

## Simple Summary

Horse fat is a traditional and nutritious food in Central Asia, used in dishes passed down through generations. Because horse fat contains a high proportion of beneficial unsaturated fats, it spoils more quickly than other animal fats—a problem that is poorly understood and rarely studied. This study examined how quickly raw horse fat taken from the abdominal area deteriorates when stored under warm, humid conditions typical of summer markets and transport in the region. Fat samples from ten horses were stored at 30 °C with 70% relative humidity for six days, and their quality was measured on days zero, three, and six using chemical tests and an infrared light technique that detects changes in the fat’s molecular structure. The results showed that the fat deteriorated rapidly, exceeding internationally accepted safe limits for free fatty acid content within six days, and became completely unacceptable to the senses—turning yellow, softening, and developing a strong rancid smell. These findings highlight the urgent need for cold-chain management and proper refrigeration when handling and selling horse fat products in Central Asia, to protect consumers and preserve the quality of this culturally important food.

## 1. Introduction

Lipid oxidation is one of the primary deterioration mechanisms in animal fats, and its rate is strongly dependent on the degree of fatty acid unsaturation [1]. Fundamentally, it proceeds via a free-radical chain reaction between unsaturated fatty acids and molecular oxygen, yielding hydroperoxides as the primary oxidation products. Although these initial compounds are odorless and do not directly affect aroma, their inherent instability leads to rapid decomposition. This degradation generates a diverse array of secondary compounds including hydrocarbons, aldehydes, ketones, alcohols, esters, and acids [2] which are ultimately responsible for the development of off-flavors and undesirable odors in meat. Among degradation processes in meat and meat products, oxidative processes are considered the most important after microbial deterioration, affecting lipids, pigments, proteins, and vitamins, leading to sensory degradation and nutritional loss, and potentially forming toxic substances [3]. Horse fat is distinguished by its exceptionally high PUFA content compared to other animal fats, making it particularly susceptible to rapid oxidative deterioration. Horse fat is a valuable dietary product, rich in unsaturated fatty acids, with the total content of ⍵-3 polyunsaturated fatty acids (PUFAs) in muscle fat ranging from 1.17% to 18.9%. Pasture grazing has pronounced impacts on fatty acid composition in monogastric animals such as horses, with major fatty acids including palmitic (24.85%), oleic (27.34%), linolenic (17.95%), linoleic (11.20%), and stearic (5.54%) acids [4,5]. Fat tissues around the heart and abdominal layer were found to be more saturated than mesenteric, intermuscular, and subcutaneous adipose tissues, meaning that mesenteric (visceral) fat carries a comparatively higher proportion of unsaturated fatty acids and is therefore among the most oxidatively labile fat depots [6]. Mesenteric fat was selected as the focus of this study because it is both the most oxidatively labile of the major equine adipose depots, owing to its comparatively higher unsaturated fatty acid content relative to cardiac, abdominal and subcutaneous fat [6], and because it is among the depots most commonly collected, traded and consumed raw in traditional Central Asian food practice, making it the most representative matrix for assessing food-safety-relevant deterioration kinetics under market storage conditions. Lipid oxidation in meat and meat products is generally described as a free-radical chain reaction initiated by reactive oxygen species, leading to the formation of lipid hydroperoxides and secondary oxidation products [7]. Fourier-transform infrared (FTIR) spectroscopy has emerged as a powerful tool for monitoring lipid oxidation in animal tissues. Studies on foal meat have demonstrated that ATR-FT/MIR (Mid-Infrared) spectroscopy can reliably establish prediction models for lipid and protein [8] oxidation marker compounds, providing a rapid and non-destructive analytical approach.

Raw horse mesenteric adipose tissue is a valuable by-product appreciated by consumers in Central Asia, where this fat is regarded as a food with nutritional and dietetic interest. The fatty acid composition of mesenteric, cardiac, abdominal, intermuscular, and subcutaneous adipose tissues from horses has been described in the scientific literature, establishing the nutritional significance of these depots [6]. In Kazakhstan and neighboring countries (Uzbekistan, Kyrgyzstan), horse fat occupies a central place in traditional cuisine—it is used in dishes such as qazy, qarta, and beshbarmak (qazaqsha et)—reflecting centuries of nomadic food culture [9]. Recent region-specific studies indicate that horse meat and horse-fat-containing traditional products remain important in Kazakhstan and Central Asia, but their quality is highly dependent on raw-material handling, fat addition, packaging, storage temperature and market hygiene. In a recent Kazakhstan study on traditional molded smoked ham produced from horse and camel meat, chilled raw materials were processed 48 h post mortem, while 10–20% horse fat was added to prevent dryness and improve juiciness and flavor; the finished vacuum-packed product was stored for 30 days at 0–4 °C. During storage, lipid deterioration was evident: acid value increased by about 99% in the control sample, peroxide value increased significantly by day 30, and TBARS rose from 0.16/0.12 mg MDA/kg on day 1 to 0.67/0.68 mg MDA/kg on day 30, although microbial indicators, including coliforms and Salmonella spp., were not detected under controlled processing conditions [10]. In Xinjiang Kazakh smoked horsemeat sausages, horse lean meat and back fat were mixed with 2–2.5% salt and 1.5–2% sugar, smoked and air-dried/naturally fermented for 30–40 days, with moisture, water activity, nitrite and pH identified as key factors shaping microbial communities and product stability [11].

Despite its cultural significance and widespread consumption in Central Asia, the oxidative stability and physicochemical changes in raw horse mesenteric adipose tissue under sub-optimal storage remain insufficiently characterized. Previous studies on horsemeat and equine fat have shown that prolonged storage increases acid and peroxide values, reflecting the progressive development of hydrolytic and oxidative rancidity. Because horse fat contains a high proportion of unsaturated fatty acids, approximately 57–58% of total fatty acids, it is particularly prone to oxidation, especially at elevated temperatures. Primary oxidation products may further decompose into secondary compounds, such as aldehydes, ketones, and organic acids, which are major contributors to quality loss in animal fats. However, the reaction order and rate constants associated with these processes in horse mesenteric fat have not yet been established. Quantitative evaluation of these parameters is essential for evidence-based shelf-life prediction and cold-chain management for this product category [12]. Therefore, the present study investigated the degradation kinetics of raw horse mesenteric fat obtained from ten free-grazing animals in Northern Kazakhstan. To simulate unfavorable transportation and open-market handling conditions typical of Central Asian summer climates, samples were stored at 30 °C and 70% relative humidity for six days. Physicochemical and spectroscopic analyses were performed on days 0, 3, and 6. Application of elevated temperature and humidity to accelerate deterioration within a feasible experimental timeframe is a well-established approach in lipid stability research [13]. While previous studies have characterized either the fatty acid composition of horse adipose depots [4,6] or the oxidative behavior of rendered/processed horse fat under extended high-temperature storage [7], no study to date has quantified the deterioration kinetics—including reaction order, rate constant, and the concurrent sensory, physicochemical and FTIR-spectroscopic trajectory—of raw, unprocessed mesenteric fat under conditions representative of short-term ambient market storage. This distinction is critical, as raw mesenteric fat retains endogenous lipolytic activity absent in rendered fat, and is consumed and traded in this raw form throughout Central Asia. Acid value, peroxide value, refractive index, density, melting point, and ATR-FTIR spectral profiles were assessed at each time-point, and kinetic modeling of acid value progression was performed to determine the reaction order and rate constant of lipid deterioration in this matrix. The aim of this study was to evaluate the deterioration kinetics of raw horse mesenteric fat under accelerated storage conditions using physicochemical, sensory, and FTIR-ATR analyses.

## 2. Materials and Methods

### 2.1. Sampling and Experimental Conditions

Raw horse mesenteric adipose tissue of 10 different free-grazing animals was sourced from the northern part of Kazakhstan (coordinates: 50.965563° N, 71.352382° E). Ethical approval was waived by the Institutional Ethics Committee of LLP NPP Antigen (Almaty, Kazakhstan), as the study exclusively utilized post-mortem adipose tissue collected from animals slaughtered as part of routine commercial meat production under standard husbandry practices. No experimental procedures were performed on live animals, and no animals were sacrificed specifically for this study. All animals were free-grazing horses (age range 4–9 years; both sexes represented) raised on natural pasture without supplementary feeding. Slaughter was performed at a licensed commercial abattoir under standard hygienic conditions, and adipose tissue samples were collected and transferred to frozen storage (−20 °C) within 2 h. All analyses were performed within 5 days of sample collection to minimize enzymatic and oxidative changes. Prior to the experiment, the fat was portioned into 300 g samples of each animal. The accelerated aging process was conducted using a high-precision constant HPP260eco constant climate chamber (Memmert GmbH + Co. KG, Schwabach, Germany). The samples were exposed to a constant temperature of 30 °C and a relative humidity (RH) of 70%. The selected storage conditions (30 °C and 70% RH) were established with slight modification based on previously reported articles [14,15], in which elevated temperature and relative humidity were applied to accelerate oxidative reactions and enhance lipid degradation kinetics. These stress conditions were intended to simulate oxidative deterioration processes occurring during prolonged conventional storage within a significantly reduced experimental timeframe. The evaluation of the samples was performed on days 0, 3, and 6 of the storage. Prior to the start of the storage trial, frozen samples were thawed at 4 °C for 12 h under refrigerated conditions. Day 0 measurements were performed immediately following this controlled thawing step and therefore represent the baseline (pre-stress-storage) condition of the fat, rather than the originally fresh, pre-freezing state. Each of the 10 animals constituted an independent biological replicate (*n* = 10). For each animal and each time-point, all physicochemical measurements were additionally performed in technical duplicate, and the mean of the two technical replicates was used as the single value representing that animal at that time-point for subsequent statistical analysis.

### 2.2. Methods

The Ash of fats was measured by Marshall [16], which involves the complete thermal destruction of organic matter. The sample was incinerated and ignited in a muffle furnace at 600 °C temperatures (SNOL 7,2/1200, Utena, Lithuania) until a constant mass of inorganic residue was obtained.

Sensory evaluation was conducted in accordance with ISO 6658:2017 [17]. The organoleptic quality of horse mesenteric adipose tissue was assessed at each sampling point (days 0, 3, and 6) by a panel of three trained evaluators. All panelists had prior experience in the sensory assessment of animal fats and meat products and completed a calibration session using reference samples with known oxidative status before the study. Evaluations were performed under white light at room temperature (22–24 °C) in a dedicated, odor-neutral assessment room. Each of the 10 individual animal samples was coded and presented to the panelists in randomized order to ensure blinding with respect to storage day. The evaluated attributes included visible color, consistency at room temperature, and odor, which are commonly used indicators of lipid quality and oxidative deterioration. Each attribute was rated using a five-point descriptive scale, where a score of 5 represented characteristics typical of fresh adipose tissue and a score of 1 indicated severe quality deterioration (Table 1). For each animal and time point, samples were assessed independently by all three panelists, and the final score for each attribute was calculated as the mean of the three independent evaluations.

The refractive index (RI) was determined using a refractometer IRF-454B2M (Kazan Optical-Mechanical Plant JSC [KOMZ], Kazan, Russia). For the analysis, aliquots of the melted fat fraction were taken directly from the samples stored in the climate chamber.

Acid Value (AV) was determined by titration according to ISO 660:2020 [18]. The value was calculated using the following equation:

$$I_{A}=\frac{V\cdot 5.611}{a}$$

where *V* is the volume (mL) of 0.1 M sodium hydroxide or 0.1 M potassium hydroxide solution consumed during titration; *a* is the mass of the test sample (5 ± 2 g); and 5.611 is the factor corresponding to the amount of potassium hydroxide (mg) equivalent to 1 mL of 0.1 M NaOH solution. Results are expressed in mg KOH per gram of fat.

The peroxide value (PV) was determined by iodometric titration according to ISO 3960:2017 [19]. The value was calculated as follows:

$$I_{P}=\frac{1000\cdot (V-V_{0})\cdot c}{a}$$

where *V* is the volume (mL) of 0.01 M sodium thiosulfate (JSC Scientific Production and Investment Enterprise “Uralhiminvest”, Ufa, Russia) solution consumed in the main experiment; *V*_0_ is the volume (mL) of 0.01 M sodium thiosulfate consumed in the blank control; *a* is the mass of the test sample (5 ± 2 g); and *c* is the molar concentration of the sodium thiosulfate solution (mol/L). Results are expressed in millimoles of active oxygen per kilogram of fat (mmol O_2_/kg). Both parameters were measured on days 0, 3, and 6 of accelerated storage. All measurements were performed in duplicate and results reported as mean ± standard deviation.

The density of horse fat samples (5 ± 1 g) was measured using a semi-electronic density meter LSD-300 (Qunlong, Xiamen, China). The measurement is based on the Archimedes principle: each sample was weighed twice—first in air, then submerged in distilled water—and the density was calculated as

$$\rho =\frac{mair}{mair-mwater}\times \rho \;water$$

where

- m air—sample weight in air (g);
- m water—apparent sample weight placed fully in water (g); recorded as a negative value, since fat is less dense than water and exerts an upward buoyant force;
- *ρ* water—density of distilled water at the measurement temperature (g/cm^3^); value used: 0.9982 g/cm^3^.

Each sample was measured twice, and the mean value was recorded.

The melting point (MP) of horse fat was determined using a melting point apparatus (GY-30, Shanghai Zhuoguang Instrument Co., Ltd., Shanghai, China) by the capillary method. A small amount of fat was injected into a glass capillary tube (d 1 mm), and the tube was placed in the apparatus. The temperature was raised at a controlled rate by 0.5 °C; the initial melting temperature (T1) and the complete melting temperature (T2) were recorded visually. The melting point for each replicate was taken as

$$Tmp=\frac{T1+T2}{2}$$

where

- T1—temperature at which the fat begins to melt (°C);
- T2—temperature at which the fat is completely liquefied (°C).

Three independent replicates were performed per sample, and the mean melting point was recorded.

The Fourier-transform infrared (FTIR) spectroscopy was performed using an IRSpirit-TX spectrometer (Shimadzu Corporation, Kyoto, Japan) equipped with an attenuated total reflectance (ATR) module. Spectra were collected over the wavenumber range of 4500–400 cm^−1^ at a spectral resolution of 2 cm^−1^. To enhance spectral reliability and improve the signal-to-noise ratio, each spectrum represented the average of 45 successive scans. Background spectra were acquired under identical instrumental conditions prior to each measurement and automatically subtracted from the corresponding sample spectra. The obtained spectral data were subsequently processed by baseline correction and normalization procedures using LabSolutions IR software (Shimadzu Corporation, Kyoto, Japan). FTIR spectroscopy was utilized to characterize structural and compositional modifications associated with lipid oxidation and degradation during accelerated storage. For interpretation, peak position (cm^−1^) and corrected intensity were selected as the primary parameters, as corrected intensity accounts for baseline variation and provides a more reliable reflection of true absorbance changes associated with structural modifications of the lipid matrix. The functional group assignments for the observed absorption bands are summarized in Table 2.

#### Kinetic Modeling of Acid Value Deterioration

The kinetic order of acid value deterioration was determined by fitting the mean AV data to zero-order and first-order kinetic models. For zero-order kinetics, AV was plotted linearly against storage time: C(t) = C_0_ + k·t. For first-order kinetics, the natural logarithm of AV was plotted against storage time: ln(C(t)) = ln(C_0_) + k·t. The model with the higher coefficient of determination (R^2^) was selected as the best fit. The rate constant k was derived from the slope of the linearized regression. Regression fit was compared using R^2^: zero-order R^2^ = 0.927 (k = 5.237 mg KOH/g·day^−1^); first-order R^2^ = 0.959 (k = 0.554 day^−1^).

### 2.3. Statistical Analyses

To compare the different physicochemical values (RI, AV, PV, density and MP) at different days, the non-parametric test (Friedman test of k series of samples with the procedure of Nemenyi) was used because of the heteroscedasticity of the data [25]. The test was completed by a multiple pairwise comparisons following Dunn’s procedure (bilateral test) [26]. Similar analysis was achieved to assess the differences in the area for the different functional groups in FTIR. The results were expressed by mean ± SD. Level of significance threshold was *p* < 0.05. The software used was XLstat version 2026 (© Addinsoft, Paris, France).

The authors disclose that generative artificial intelligence (Gemini 3 Pro) was utilized during the preparation of this manuscript. The use of these tools was strictly confined to text structuring, clarity enhancement, and conceptual visualization. All scientific data collection, experimental analyses, interpretation of results, and final manuscript verification were performed solely by the authors, who maintain full responsibility for the content and integrity of the published work.

## 3. Results

The experimental duration of six days was determined based on the complete sensory failure of all samples by day 6, as evidenced by strong rancid odor, pronounced discoloration (transition from white to bright yellow with visible dark spots), and overall organoleptic unacceptability. Sensory evaluation confirmed organoleptic failure by day 6, with mean scores for color, consistency and odor falling below the predefined acceptability threshold (score < 3.0). According to the literature, it is well established that, for highly perishable food products, the end of shelf life is primarily determined by the loss of sensory attributes or the appearance of organoleptic defects readily recognizable by the consumer, such as off-odors and discoloration, rather than by nutritional loss criteria applied to longer-shelf-life products [27]. The six-day experimental duration was determined by complete sensory failure of all samples, evidenced by strong rancid odor, pronounced discoloration, and overall organoleptic unacceptability (mean score < 3.0 across all attributes; Table 3). Storage was terminated at this point, consistent with established shelf-life testing practice in which the study endpoint is defined by the first failure criterion reached, whether sensory or chemical [27,28,29]. The Ash of initial fat was 0.35%. All the results regarding the physicochemical parameters were summarized in Table 4.

The refractive index of horse fat showed only minor variations during accelerated oxidation under climatic chamber conditions. Initial refractive index values remained stable at 1.470 on both day 0 and day 3, followed by a slight decrease to 1.460 at day 6. Similarly, the minimum refractive index decreased from 1.465 to 1.462 over the storage period, whereas the maximum values showed only slight fluctuations (1.467–1.468) (Table 4). These results suggest that the refractive index is relatively stable during the early stages of lipid oxidation, although prolonged thermal exposure may induce minor structural alterations in the lipid matrix associated with oxidative degradation of unsaturated fatty acids.

The acid value of horse fat increased markedly and significantly during accelerated oxidation under climatic chamber conditions, indicating progressive hydrolytic and oxidative degradation of the lipid matrix. A substantial increase in standard deviation was also observed over the storage period, rising from 0.459 at day 0 to 24.915 and 26.183 at days 3 and 6, respectively. This elevated variability suggests non-uniform progression of lipid degradation among samples during prolonged thermal exposure. The pronounced increase in acid value reflects the accumulation of free fatty acids resulting from triglyceride hydrolysis and oxidative decomposition, confirming the high susceptibility of horse fat to rancidity under elevated temperature and humidity conditions [30,31]. The first-order model provided the marginally better fit (R^2^ = 0.959 vs. 0.927), indicating that the rate of free fatty acid accumulation was proportional to the current acid value—a pattern consistent with autocatalytic lipid oxidation, in which initial hydrolysis and oxidation products promote subsequent degradation reactions [30]. However, both models describe the data adequately, and given the limited number of time-points (*n* = 3), these kinetic parameters should be interpreted as indicative rather than definitive [32].

The peroxide value of horse fat increased progressively during accelerated storage in the climatic chamber, indicating the formation and accumulation of primary lipid oxidation products. This trend reflects increasing variability in oxidative stability among the samples during prolonged exposure to elevated temperature and humidity. The increase in peroxide value is associated with the generation of lipid hydroperoxides, which are recognized as the primary products of lipid oxidation and are commonly used as indicators of the early stages of oxidative deterioration in fats and oils [30,33].

The initial decline in density on day 3 may be attributed to the thermal softening and partial melting of fat fractions under elevated temperature conditions, leading to structural loosening of the lipid matrix. The decline in density from day 0 to day 3 may be attributed to thermal softening and partial melting of fat fractions under elevated temperature conditions, leading to structural loosening of the lipid matrix. The further decrease from day 3 to day 6 occurred concurrently with the continued rise in acid value, consistent with a progressive change in lipid matrix composition; however, in the absence of compositional or volatile analysis, the specific molecular basis of this change cannot be established from the present data and should be investigated through fatty acid profiling in future work.

On day 0, the mean *melting point* was 32.145 ± 4.331 °C, reflecting considerable variability among individual animals, which is characteristic of raw mesenteric adipose tissue due to natural biological variation in fatty acid composition between animals. By day 3, the melting point decreased significantly to 25.948 ± 0.486 °C, with a notably reduced standard deviation, suggesting a convergence in the lipid thermal behavior across samples. A slight recovery was observed on day 6, with a mean value of 26.929 ± 0.993 °C. The pronounced decrease in melting point between days 0 and 3 may be associated with oxidative and hydrolytic modification of triglycerides and changes in lipid crystal organization under the applied stress conditions. As fatty acid profiling was not performed at each time-point in this study, we cannot directly attribute this thermal shift to a specific change in chain length or unsaturation, and this should be considered an avenue for confirmation in future work incorporating GC-FID analysis at each storage interval. Additionally, partial hydrolysis of triglycerides, as reflected by the concurrent increase in acid value, may contribute to the disruption of the crystalline lipid network, further lowering the thermal transition point [34]. Oxidative degradation of lipid molecules is known to alter the physical and thermal properties of fats, including melting behavior and crystal structure [35]. The marginal increase observed on day 6 (26.929 °C) could reflect the accumulation of secondary oxidation products and polymerization of oxidized lipid species, which tend to increase molecular weight and restore partial structural ordering within the fat matrix. The substantially reduced standard deviation from day 3 onward (≤1.0 °C) indicates progressive homogenization of the lipid fraction under oxidative stress conditions, with inter-animal variability becoming negligible as oxidative deterioration proceeds [29]. These findings suggest that the melting point is a sensitive indicator of early-stage lipid modifications in raw horse fat and may serve as a complementary parameter alongside chemical oxidation markers in shelf-life assessment studies.

**Figure 1:**
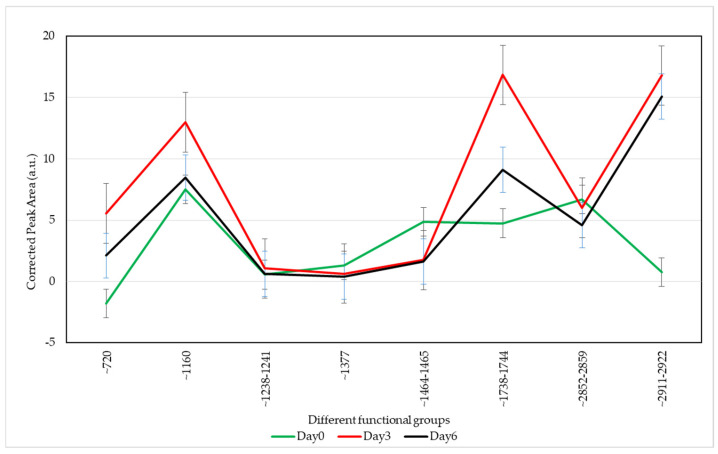
Changes in the functional groups at different wavenumbers in horse mesenteric fat at day 0, 3 and 6 under accelerated storage (mean and SD).

FTIR Spectroscopy results of one of the most analytically significant changes across the storage period were observed in the carbonyl region (~1738–1744 cm^−1^) and the C–H stretching region (~2852–2922 cm^−1^). On day 0, no distinct carbonyl peak was resolved in the mean spectrum, whereas by day 3 a prominent absorption band appeared at 1743.6 cm^−1^ (corrected intensity: 0.739), which slightly decreased by day 6 (1738.8 cm^−1^; corrected intensity: 0.462) (Figure 1). The emergence of this carbonyl band is consistent with the accumulation of oxidative carbonyl-containing compounds, including aldehydes and ketones, which are characteristic secondary oxidation products formed during lipid peroxidation. The subsequent decrease in corrected intensity on day 6 may reflect further decomposition of these intermediates into volatile low-molecular-weight products or their participation in secondary polymerization reactions.

In the C–H stretching region, the corrected intensity of the asymmetric CH_2_ stretching band (~2920–2922 cm^−1^) increased from day 0 (0.031) to day 3 (0.636) and remained elevated on day 6 (0.573), suggesting progressive conformational changes in the fatty acid acyl chains associated with oxidative degradation of the lipid matrix. Similarly, the symmetric CH_2_ stretching band (~2852–2859 cm^−1^) showed increased corrected intensity from day 3 onward (0.463 and 0.388 on days 3 and 6, respectively), further supporting chain-level structural modification (Table 5).

The band at ~1160 cm^−1^, attributed to C–O ester stretching in triglycerides, exhibited increased corrected intensity over the storage period (0.181 on day 0; 0.311 on day 3; 0.207 on day 6), which may reflect partial hydrolysis of ester bonds and the concomitant release of free fatty acids, consistent with the observed increase in acid value. The CH_2_ rocking band at ~720 cm^−1^ showed fluctuating corrected intensity values (0.104 → 0.201 → 0.154), indicating changes in the conformational ordering of methylene chains, potentially linked to phase transitions and structural reorganization of the lipid fraction under the applied thermal and humidity stress.

**Figure 2:**
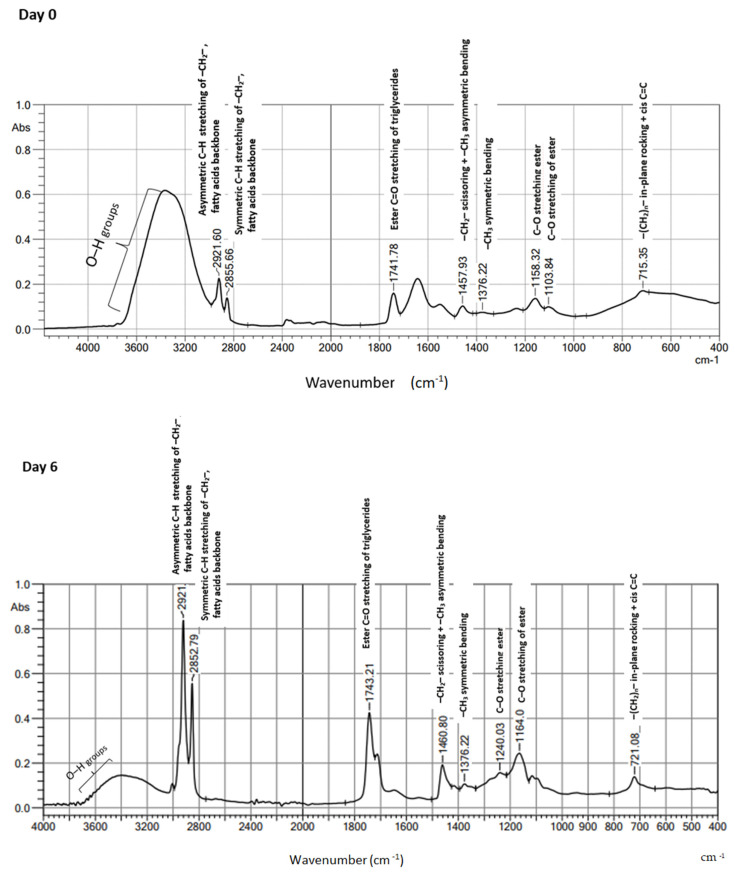
The MIR spectrum corresponding to lipid oxidation compounds in 0 and 6 days.

Collectively, the FTIR data confirm progressive oxidative and hydrolytic deterioration of horse mesenteric fat during accelerated storage, with the carbonyl region (~1738 cm^−1^) and C–H stretching bands serving as the most sensitive spectral markers of lipid degradation kinetics (Figure 2).

## 4. Discussion

The rapid deterioration of raw horse mesenteric fat observed in the present study is consistent with its high content of polyunsaturated fatty acids, which are the primary substrates for free-radical lipid oxidation. Specifically, the abundance of linoleic acid (C18:2n-6) and α-linolenic acid (C18:3n-3) in equine adipose tissue provides multiple positions highly susceptible to hydrogen abstraction by reactive oxygen species, a structural vulnerability not shared by the more saturated fat depots of ruminant species such as cattle or sheep [30]. This compositional difference likely explains why horse fat exhibits a markedly shorter shelf life than tallow or lard under comparable storage conditions, a point that merits explicit emphasis given the practical implications for traditional Central Asian food handling practices.

The pronounced increase in acid value, exceeding the Codex Alimentarius threshold of 2.0 mg KOH/g by day 3, reflects accelerated triglyceride hydrolysis under the applied conditions of 30 °C and 70% RH, and is in agreement with previous reports demonstrating that elevated temperature storage markedly accelerates free fatty acid accumulation in equine adipose tissue [30,31]. Mechanistically, this hydrolysis is most plausibly driven by residual endogenous lipases retained in the raw, unrendered tissue, since no exogenous microbial inoculum was introduced; this is consistent with reports that raw animal fats, unlike rendered or refined fats, retain measurable lipolytic enzyme activity post-slaughter that continues to act under ambient storage [31]. The high relative humidity (70%) applied in this study likely further accelerated hydrolysis by providing the aqueous phase required for ester bond cleavage, a factor that should be considered separately from the thermal effect in future kinetic modeling.

The autocatalytic character of this process, supported by the marginally better fit of the first-order kinetic model (R^2^ = 0.959) compared with the zero-order model (R^2^ = 0.931), indicates that initial hydrolysis and oxidation products catalyze subsequent degradation reactions, a mechanism that is well established for lipid-rich food matrices [30,32]. Free fatty acids generated during early hydrolysis are themselves more oxidatively labile than esterified fatty acids, creating a feed-forward loop in which hydrolysis promotes oxidation and oxidation products in turn promote further hydrolysis through localized acidification—a synergistic mechanism that may explain why degradation accelerates rather than plateaus between days 3 and 6.

The non-significant increase in peroxide value, despite the marked rise in acid value, suggests that primary oxidation products (hydroperoxides) were rapidly decomposing into secondary carbonyl-containing compounds, a process corroborated by the emergence and subsequent decline of the FTIR carbonyl band at ~1743 cm^−1^ between days 3 and 6 [33]. This apparent discrepancy between a stable peroxide value and visibly progressing spoilage is of particular practical relevance, as it implies that peroxide value alone is an unreliable indicator of spoilage in raw horse fat and should not be used in isolation for quality control or shelf life labeling—acid value and FTIR carbonyl signals provide more sensitive and temporally consistent markers for this specific matrix.

The decline in density and melting point observed from day 0 to day 3 is consistent with preferential hydrolytic cleavage of higher-molecular-weight saturated triglycerides, yielding a lipid matrix progressively enriched in shorter-chain and unsaturated species [34,35]. The partial recovery of melting point on day 6 may reflect the onset of polymerization of oxidized lipid species, a phenomenon associated with increased molecular weight and partial restoration of structural order in heavily oxidized fats [35]. This non-monotonic thermal behavior is noteworthy because it indicates that melting point cannot be used as a simple, unidirectional shelf life marker for this product—a practical caveat directly relevant to any future attempt to develop rapid field-deployable quality assessment tools for traditional markets.

The large inter-animal variability in acid value, particularly evident from day 3 onward, most likely reflects biological heterogeneity in the initial fatty acid composition of mesenteric fat among individual horses, which is known to vary substantially with grazing conditions and body condition score [6]. This variability has a direct practical consequence: a single, universal shelf life recommendation may be inappropriate for horse fat sourced from heterogeneous, pasture-raised populations, and risk-based or batch-specific quality testing may be necessary to ensure consumer safety across different production contexts.

Collectively, the physicochemical and FTIR-ATR data demonstrate that raw horse mesenteric fat reaches organoleptic and chemical failure thresholds within three to six days under conditions representative of Central Asian summer markets, underscoring the urgent need for effective cold-chain management and evidence-based shelf life guidelines for this traditionally consumed product. These findings collectively support the use of acid value and FTIR carbonyl signal as primary, complementary indicators for shelf life determination of raw horse fat, while highlighting peroxide value and melting point as secondary, context-dependent parameters requiring cautious interpretation.

## 5. Conclusions

The aim of this study was to evaluate the deterioration kinetics of raw horse mesenteric fat under accelerated storage conditions using physicochemical, sensory, and FTIR-ATR analyses. The results showed that raw horse mesenteric fat deteriorated rapidly at 30 °C and 70% RH, reaching unacceptable chemical and sensory quality thresholds within three to six days. Acid value increased markedly and was best described by a first-order kinetic model, indicating rapid accumulation of free fatty acids during storage. FTIR-ATR analysis confirmed concurrent structural changes in the lipid matrix, particularly in the aliphatic C–H stretching, ester carbonyl C=O stretching, and C–O stretching regions.

These findings indicate that acid value, supported by FTIR-ATR spectroscopy, can serve as a practical approach for monitoring early deterioration of raw horse fat. The results also highlight the need for strict cold-chain management and evidence-based handling guidelines for horse fat products in Central Asia. However, FTIR-ATR does not allow direct identification of specific secondary oxidation compounds. Therefore, future studies should include fatty acid profiling, microbiological analysis, and complementary chromatographic methods such as GC–MS or LC–MS to confirm degradation products and improve shelf-life prediction under real storage conditions.

## Figures and Tables

**Table 1:** Sensory evaluation scoring scale for horse mesenteric adipose tissue during accelerated storage.

Score	Color	Consistency	Odor
5	White to pale yellow, uniform	Firm, homogeneous	Fresh, neutral, characteristic of raw fat
4	Slight yellowing	Slightly softened	Mild, barely perceptible off-note
3	Noticeable yellowing	Moderately soft, slightly greasy surface	Detectable rancid or sour note
2	Yellow to light brown	Soft, oily, surface discoloration	Distinct rancidity
1	Brown or gray tones, uneven	Liquid at room temperature, phase separation	Strong rancid, putrid odor

**Table 2:** Assignment of FTIR absorption bands identified in horse mesenteric fat spectra.

Wavenumber (cm^−1^)	Assignment	Reference
~2911–2922	CH_2_ asymmetric stretching/Methylene groups of fatty acid alkyl chains	[20]
~2852–2859	CH_2_ symmetric stretching/Methylene groups of fatty acid alkyl chains	[20]
~1738–1744	C=O stretching (ester carbonyl)/Triglyceride ester carbonyl; aldehydes and ketones from secondary oxidation	[21]
~1464–1465	CH_2_ scissoring/CH_3_ asymmetric bending/Saturated fatty acid methylene chains	[22]
~1377	CH_3_ symmetric bending/Methyl end groups of fatty acid acyl chains	[23]
~1238–1241	C–O–C asymmetric stretching/Ester groups in glycerol backbone of triglycerides	[20]
~1160	C–O stretching/Ester linkage in triglycerides (C–O–C asymmetric stretch)	[24]
~720	CH_2_ rocking/Long-chain methylene sequences; characteristic of cis-disubstituted C=C in unsaturated fatty acids	[20]

**Table 3:** Sensory evaluation scores (mean ± SD, *n* = 10 animals) of horse mesenteric adipose tissue during accelerated storage at 30 °C/70% RH.

Attribute	Day 0	Day 3	Day 6
Color	5.0 ± 0.0	3.3 ± 0.6	1.7 ± 0.6
Consistency	5.0 ± 0.0	3.7 ± 0.6	2.3 ± 0.6
Odor	5.0 ± 0.0	2.7 ± 0.6	1.0 ± 0.0
Overall	5.0	3.2	1.7

**Table 4:** Physicochemical changes in horse mesenteric fat at day 0, day 3 and day 6 under accelerated storage (mean and SD).

Parameter	Day 0	Day 3	Day 6	*p*
Refractive index	1.4662 ± 0.0004 ^a,b^	1.4667 ± 0.00075 ^a^	1.4634 ± 0.00147 ^b^	<0.0001
AV (mg KOH/g)	1.1721 ± 0.4593 ^a^	20.3118 ± 24.9154 ^a,b^	32.5973 ± 26.1832 ^b^	<0.0001
PV (mmol O_2_/kg)	4.2691 ± 5.9229 ^a^	6.3845 ± 6.6866 ^a,b^	8.7582 ± 7.4602 ^b^	<0.001
Density (g/cm^3^)	0.9333 ± 0.0283 ^a^	0.9021 ± 0.0201 ^a^	0.8932 ± 0.0333 ^a^	NS
Melting point (°C)	32.145 ± 4.3311 ^a^	25.948 ± 0.486 ^b^	26.929 ± 0.9933 ^b^	<0.0001

*^a,b^ The different subscripts in the same line differs at p < 0.05. NS—not significant.*

**Table 5:** Area values (mean and SD) of the functional groups at different wavenumbers in horse mesenteric fat at day 0, 3 and 6 under stress storage.

Wavenumber (cm^−1^)	Day 0	Day 3	Day 6	*p*-Value
~720	53.678 ± 33.85 ^b^	29.951 ± 17.93 ^a^	43.181± 28.5 ^a,b^	**0.027**
~1160	24.521 ± 17.32 ^a^	36.452 ± 10.81 ^a^	28.701 ± 14.15 ^a^	0.236
~1238–1241	7.780 ± 6.06 ^a^	16.614 ± 6.48 ^b^	11.740 ± 7.57 ^a,b^	**0.006**
~1377	6.810 ± 5.45 ^a^	6.468 ± 2.43 ^a^	4.923 ± 2.61 ^a^	0.273
~1464–1465	9.058 ± 9.56 ^a^	7.401 ± 4.15 ^a^	8.099 ± 4.63 ^a^	0.497
~1738–1744	10.184 ± 9.36 ^a^	24.642 ± 7.57 ^b^	19.805 ± 9.79 ^a,b^	**0.002**
~2852–2859	15.312 ± 13.62 ^a^	17.850 ± 5.17 ^a^	18.197 ± 6.99 ^a^	0.202
~2911–2922	5.625 ± 0.0 ^a^	33.077 ± 9.50 ^b^	33.007 ± 10.14 ^b^	**0.001**

*^a,b^ The different subscripts in the same line differs at p < 0.05.*

## Data Availability

The data supporting the findings of this study are available within the article.

## Abbreviations

The following abbreviations are used in this manuscript:

AV	Acid Value
PV	Peroxide Value
PUFA	Polyunsaturated Fatty Acid
FTIR	Fourier-Transform Infrared Spectroscopy
ATR	Attenuated Total Reflectance
MIR	Mid-Infrared
RI	Refractive Index
RH	Relative Humidity
SD	Standard Deviation
NS	Not Significant
